# Reactive oxygen species and p47phox activation are essential for the *Mycobacterium tuberculosis*-induced pro-inflammatory response in murine microglia

**DOI:** 10.1186/1742-2094-4-27

**Published:** 2007-11-26

**Authors:** Chul-Su Yang, Hye-Mi Lee, Ji-Yeon Lee, Jeong-Ah Kim, Sung Joong Lee, Dong-Min Shin, Young-Ho Lee, Dong-Seok Lee, Jamel El-Benna, Eun-Kyeong Jo

**Affiliations:** 1 Department of Microbiology, College of Medicine, Chungnam National University, Daejeon 301-747, S. Korea; 2 Department of Anatomy, College of Medicine, Chungnam National University, Daejeon 301-747, S. Korea; 3 Department of Oral Physiology, School of Dentistry, Seoul National University, 28 Yeongun-dong, Jongno-gu, Seoul 110-749, S. Korea; 4 College of animal resource sciences, Kangwon National University, Chunchon 200-701, Korea; 5 Inserm U773, Université Paris 7-Denis Diderot, Site Bichat, Paris, France; 6 Infection Signaling Network Research Center, College of Medicine, Chungnam National University, Daejeon 301-747, S. Korea

## Abstract

**Background:**

Activated microglia elicits a robust amount of pro-inflammatory cytokines, which are implicated in the pathogenesis of tuberculosis in the central nervous system (CNS). However, little is known about the intracellular signaling mechanisms governing these inflammatory responses in microglia in response to *Mycobacterium tuberculosis *(Mtb).

**Methods:**

Murine microglial BV-2 cells and primary mixed glial cells were stimulated with sonicated Mtb (s-Mtb). Intracellular ROS levels were measured by staining with oxidative fluorescent dyes [2',7'-Dichlorodihydrofluorescein diacetate (H_2_DCFDA) and dihydroethidium (DHE)]. NADPH oxidase activities were measured by lucigenin chemiluminescence assay. S-Mtb-induced MAPK activation and pro-inflammatory cytokine release in microglial cells were measured using by Western blot analysis and enzyme-linked immunosorbent assay, respectively.

**Results:**

We demonstrate that s-Mtb promotes the up-regulation of reactive oxygen species (ROS) and the rapid activation of mitogen-activated protein kinases (MAPKs), including p38 and extracellular signal-regulated kinase (ERK) 1/2, as well as the secretion of tumor necrosis factor (TNF)-α, interleukin (IL)-6, and IL-12p40 in murine microglial BV-2 cells and primary mixed glial cells. Both NADPH oxidase and mitochondrial electron transfer chain subunit I play an indispensable role in s-Mtb-induced MAPK activation and pro-inflammatory cytokine production in BV-2 cells and mixed glial cells. Furthermore, the activation of cytosolic NADPH oxidase p47phox and MAPKs (p38 and ERK1/2) is mutually dependent on s-Mtb-induced inflammatory signaling in murine microglia. Neither TLR2 nor dectin-1 was involved in s-Mtb-induced inflammatory responses in murine microglia.

**Conclusion:**

These data collectively demonstrate that s-Mtb actively induces the pro-inflammatory response in microglia through NADPH oxidase-dependent ROS generation, although the specific pattern-recognition receptors involved in these responses remain to be identified.

## Background

*Mycobacterium tuberculosis *(Mtb) infection of the central nervous system (CNS), particularly in cases of meningitis, accounts for 1 to 10% of all cases of tuberculosis (TB). It is the most severe form of systemic TB because of its high mortality rate and possible serious neurological complications. In the CNS, where the function of neurons is protected by the maintenance of an anti-inflammatory environment [1], infection with Mtb leads to catastrophic, inflammatory tissue destruction [2]. The mechanisms behind this phenomenon are currently unknown. Unlike pulmonary TB, which has been intensively investigated in numerous clinical trials, the pathogenesis, diagnosis, and treatment of CNS-TB have received little attention. A better understanding of CNS-TB pathogenesis is urgently required to improve existing therapies, which still leave over half of those affected dead or paralyzed [3].

The CNS-resident macrophages, microglia, are productively infected with Mtb and may be the principal cellular target in the CNS [4,5]. Activated microglia release a number of cytokines/chemokines that contribute to both defense against and the neuropathogenesis of CNS infection [6]. Upon activation, microglia produce and secrete potentially neurotoxic pro-inflammatory cytokines, including tumor necrosis factor (TNF)-α/β, interleukin (IL)-1 α/β, and IL-6 [7]. Both TNF-α and IL-1β have been found at increased concentrations in the cerebrospinal fluid (CSF) of patients with CNS-TB [8-10]. Upon mycobacterial infection, mitogen-activated protein kinases (MAPKs) play important roles in promoting anti-mycobacterial activity and the production of immune effector molecules, including TNF-α [11-15]. There is increasing evidence that reactive oxygen species (ROS) also function as second messengers to regulate several downstream signaling molecules, including MAPKs or the NF-κB pathway [16-18].

ROS are produced in mammalian cells in response to the activation of various cell surface receptors [17]. In brain-resident immune cells, the generation of free radicals plays important roles in anti-microbial defense as well as in pro-inflammatory signaling [19,20]. Activation of the NADPH oxidase pathway initiates an intracellular ROS signaling pathway that amplifies the production of pro-inflammatory cytokines, such as TNF-α [21]. Intracellular ROS mediate β-amyloid peptide-induced microglial activation [22]. In addition, microglia-mediated neurotoxicity is influenced by the release of microglial NADPH oxidase-mediated ROS [23-25]. Previous studies indicate that p47phox, an essential component of the phagocyte NADPH oxidase, is required for superoxide anion release from microglia [26]. To date, the roles of NADPH oxidase-derived ROS and the intracellular regulatory mechanisms by which these pro-inflammatory responses are induced in microglial cells during mycobacterial infection are poorly understood.

Activated microglia express Toll-like receptors (TLRs) [27], CD14 [4], and mannose receptors [28]. TLRs play an important role in the activation of immune cells by pathogens such as Mtb. Receptors other than TLRs, including C-type lectins, are also involved in mediating host responses to Mtb. Recently, Yadav *et al*. [29] reported that the β-glucan receptor dectin-1 works with TLR2 to mediate *Mycobacterium*-induced pro-inflammatory responses in macrophages. To date, no attempt has been made to identify the specific mycobacterial antigens that interact with specific TLRs or other pattern-recognition receptors (PRRs) in microglia. To better understand the Mtb-induced molecular signaling pathways in microglia, we selected BV-2 cell lines that retain the characteristics of activated microglial cells, and we confirmed our results in murine primary mixed glial cells. We investigated the role of ROS and MAPK signaling in the regulation of pro-inflammatory cytokine expression in response to sonicated Mtb (s-Mtb). We found that s-Mtb activates inflammatory mediators in microglial cells and primary mixed glial cells through NADPH oxidase-dependent ROS generation. In addition, p38 and extracellular signal-regulated kinase (ERK) 1/2 signaling is essential for the expression of TNF-α, IL-10, and IL-12p40 in s-Mtb-stimulated microglia. Furthermore, we investigated the potential roles of PRRs, such as TLR2 and dectin-1, in microglial cells.

## Methods

### Murine mixed glial cells, and cell lines

Mice with a targeted deletion in the *TLR2 *gene (homozygous mice and their homozygous litter mates) were kindly provided by Dr. S. Akira (Osaka University, Osaka, Japan). All animals were maintained under standard laboratory conditions on a 12-h light/dark cycle, with free access to food and water. All of the animal procedures were conducted in accordance with the guidelines of the institutional Animal Care and Use Committee, Chungnam National University.

Primary mixed glial cultures were prepared from 1- or 2-day-old neonatal C57BL/6 mice. The cerebral cortices were dissected, carefully stripped of their meninges, and digested with 0.25% trypsin for 25 min at 37°C. Trypsinization was stopped by adding an equal volume of culture medium (Dulbecco's modified Eagle medium-F-12 nutrient mixture, fetal bovine serum 10%, penicillin 100 U/mL, streptomycin 100 μg/mL, and amphotericin B (Fungizone^® ^0.5 μg/mL) to which 0.02% deoxyribonuclease I was added. The solution was pelleted, resuspended in culture medium, and brought to a single cell suspension by repeated pipetting followed by passing through a 105-μm-pore mesh. Cells (at a density of 250,000 cells/mL) were cultured at 37°C in humidified 5% CO_2_/95% air. Medium was replaced every 5–7 days. Cultures were used between 12 and 15 days *in vitro*. At this point they typically consisted of 75% type-I astrocytes and 25% microglia (data not shown). Separately, to obtain astrocyte-enriched cultures, non-astrocytes (microglia) were detached from the flasks by shaking, and removed by changing the medium [30]. We confirmed that astrocyte-enriched cultures consisted of > 95% astrocytes (data not shown).

The BV-2 murine microglial cell lines were kindly provided by Dr. Sung Joong Lee (Seoul National University, Seoul, Korea). The cells were grown and maintained in Dulbecco's modified Eagle's medium (DMEM, Gibco, U.K.) supplemented with 10% fetal bovine serum (FBS, Gibco) and 1% penicillin/streptomycin (Gibco) at 37°C in a humidified incubator under 5% CO_2_.

### Cultures of Mtb and preparation of s-Mtb

Cultures of Mtb H37Rv were grown to mid-log phase in Middlebrook 7H9 liquid medium supplemented with oleic acid/albumin/dextrose/catalase (Difco, Becton-Dickinson, Palo Alto, CA, USA), washed three times in sterile saline, and resuspended in RPMI 1640 medium at the various concentrations. Separate culture suspensions were sonicated for 10 min on ice, in order to obtain noninfective cell lysates, as described previously [31]. S-Mtb was pooled and applied to an immobilized polymyxin B column (Detoxi-Gel endotoxin removing gel; Pierce Chemical Co.). Preparations of the s-Mtb used in experiments were tested for lipopolysaccharide (LPS) contamination by a *Limulus *amebocyte lysate assay (BioWhittaker) and contained less than 50 pg/ml at the concentrations of the s-Mtb used in experiments. In order to show that the stimulatory capacity of the s-Mtb was not the result of contamination with LPS, experiments were performed with the addition of the specific LPS-inhibiting oligopeptide polymyxin B (10.0 μg/ml) before s-Mtb stimulation.

### Reagents and antibodies (Abs)

LPS (*Escherichia coli *026:B6) and peptidoglycan (PGN, *Staphylococcus aureus*) was purchased from Sigma (St. Louis, MO, USA). BLP, a synthetic bacterial lipopeptide (Pam_3_Cys-Ser-Lys_4_-OH) derived from the immunologically active N-terminus of bacterial lipoproteins, was purchased from Invitrogen (Carlsbad, CA, USA). NAC, DPI, allopurinol and rotenone were purchased from Calbiochem (San Diego, CA, USA). Dimethyl sulfoxide (DMSO; Sigma) was added to cultures at 0.1% (vol/vol) as a solvent control. Specific inhibitors of p38 MAPK, SB203580, and specific inhibitors of MEK, U0126, and PD98059 were purchased from Calbiochem. The expression plasmids that encode p47phox WT and DN, and TAT-Ser345 peptide were kindly provided by Dr. J. El-Benna (Université Paris 7-Denis Diderot, Paris, France). Cells were transfected using LipofectAMINE as indicated by the manufacturer (InvitroGen, Carlsbad, CA, USA). Specific Abs against ERK1/2, phospho-(Thr202/Tyr204)-ERK1/2, p38, phospho-(Thr180/Tyr182)-p38 Abs were purchased from Cell signalling Technology (Beverly, MA, USA). Anti-Dectin-1 mAb (clone 2A11, IgG2b) was from Serotec (Oxford, UK). Abs to p47phox (H-195), and α-actin (I-19) were purchased from Santa Cruz Biotechnology (Santa Cruz, California, USA). The anti-phospho-(Ser345)-p47phos Ab was used, as previously described  [32]. Anti-IL-1β mAb (clone 303311, IgG1) and isotype mAb were purchased from R & D system (Minneapolis, MN, USA).

### Measurement of intracellular ROS

Intracellular ROS levels were measured by 2',7'-Dichlorodihydrofluorescein diacetate (H_2_DCFDA) and dihydroethidium (DHE) assays, as previously described [32]. Briefly, BV-2 or primary mixed glial cells were stimulated with s-Mtb or LPS for 30 min. The cells were incubated with either 10 μM H_2_DCFDA or 2 μM DHE (Molecular Probes, Eugene, OR, USA) for 15 min at 37°C in 5% CO_2_. The cells were then washed and examined with a laser-scanning confocal microscope (model LSM 510; Zeiss, Germany) and the mean relative fluorescence intensity for each group of cells was measured with a Zeiss vision system (LSM510, version 2.3) and then averaged for all groups.

### Determination of NADPH oxidase activity

NADPH oxidase activities were measured by lucigenin (*bis-N*-methylacridinium nitrate) chemiluminescence assay (5 × 10^-6 ^mol/L lucigenin, Sigma) in the presence of its substrate NADPH (10^-4 ^mol/L, Sigma) as described previously [33]. In brief, BV-2 or primary mixed glial cells were incubated with s-Mtb or LPS for 30 min in the presence or absence of DPI. Lucigenin-enhanced chemiluminescence assay was performed to analyze the level of superoxide production as previously reported [33]. The cells were transferred into scintillation vials containing Krebs-HEPES buffer (100 mM NaCl, 4.7 mM KCl, 1.9 mM CaCl_2_, 1.2 mM MgSO_4_, 1.03 mM K_2_HPO_4_, 25 mM NaHCO_3_, 20 mM Na-HEPES, pH 7.4) with 5 μM lucigenin. The chemiluminescence, which occurred over the ensuing 1 min in response to the addition of 100 μM NADPH, was recorded using a luminometer (Lumet LB9507; Berthold Technologies, Bad Wildbad, Germany). The emitted light units, after subtracting a blank, were used as a measure of superoxide production. Values are expressed as relative light units per 1 × 10^5 ^cells.

### Enzyme-linked immunosorbent assay and Western blot

A sandwich enzyme-linked immunosorbent assay (ELISA) was used for detecting TNF-α, IL-6 and IL-12p40 (PharMingen, San Diego, CA, USA) in culture supernatants. Assays were performed as recommended by the manufacturers. Cytokine concentrations in the samples were calculated using standard curves generated from recombinant cytokines, and the results were expressed in picograms per milliliter.

For Western blot analysis, total cell lysates were prepared after treatment with s-Mtb or LPS during the time indicated (0~480 min). Abs to phospho-ERK1/2, phospho-p38, total ERK1/2, total p38 and α-actin were used at 1:1,000 dilutions. Membranes were developed using a chemiluminescence assay (ECL; Pharmacia-Amersham, Freiburg, Germany) and subsequently exposed to chemiluminescence film (Fuji Film, Tokyo, Japan)

### Statistical analysis

For statistical analysis, data obtained from independent experiments are presented as the mean ± SD and they were analyzed using a Student's *t *test with Bonferroni adjustment or ANOVA for multiple comparisons. Differences were considered significant for *p *< 0.05.

## Results

### S-Mtb stimulation induces intracellular ROS generation and MAPK (ERK1/2 and p38) activation in murine microglial BV-2 cells and primary cultures of mixed glial cells

**Figure 1:**
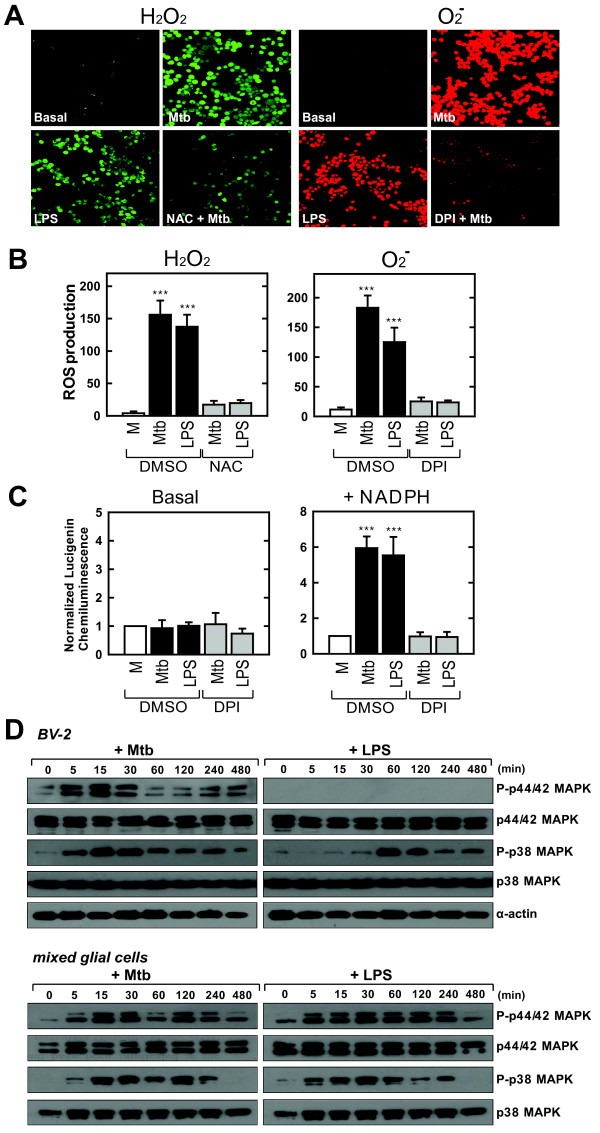
**S-Mtb induces intracellular ROS generation and MAPK (ERK1/2 and p38) activation in murine microglial BV-2 cells and in primary cultures of mixed glial cells**. *A) *BV-2 cells were incubated with DCFH-DA (H_2_O_2 _detection, left panel) or DHE (superoxide detection, right panel) in the presence or absence of 1% s-Mtb or 100 ng/ml LPS for 30 min. Live cells were washed with serum-free medium and imaged using a confocal microscope. Enhanced H_2_O_2 _or superoxide was abolished by pre-treatment with 20 mM NAC or 20 μM DPI, respectively. Images are representative of three independent experiments. *B) *Primary cultures of mixed glial cells were analyzed for H_2_O_2 _or superoxide production in response to s-Mtb or LPS treatment. The experimental conditions were identical to those outlined in *A*. Quantitative data are the mean ± SD of values from three random fields and are representative of three independent experiments. *C) *NADPH oxidase activity was quantified by measuring the production of ROS using a lucigenin-derived chemiluminescence assay. The effect of the NADPH oxidase inhibitor DPI (20 μM) was examined. M; mock. *D) *BV-2 cells (*Upper panel*) or primary mixed glial cells (*Lower panel*) were stimulated with 1% s-Mtb or 1 μg/ml LPS for the indicated times (0–480 min). The cells were then harvested and subjected to Western blot analysis to detect phosphorylated and total forms of p38 and ERK1/2. The same blots were washed and blotted for α-actin as a loading control. Data are representative of three independent experiments.

ROS may serve as intracellular signaling molecules [34]; however, ROS generation in response to mycobacterial antigens is poorly understood in microglia. We examined whether s-Mtb stimulation caused ROS generation in murine microglial BV-2 cells (Fig. 1A) and primary mixed glial cells (Fig. 1B) using the oxidative fluorescent dyes H_2_DCFDA and DHE to detect H_2_O_2 _and superoxide production, respectively. LPS treatment activated ROS generation in microglia. The chemiluminescent signal intensities attributable to H_2_O_2 _and superoxide production increased markedly in BV-2 microglial cells stimulated with s-Mtb within 30 min (data not shown). The antioxidant NAC and the NADPH oxidase inhibitor DPI significantly attenuated s-Mtb-induced H_2_O_2 _and superoxide production (Fig. 1A and 1B). When NADPH oxidase activity was measured in cultured microglial BV-2 cells via lucigenin chemiluminescence, the s-Mtb-stimulated cells showed increased NADPH oxidase activity compared to resting cells (Fig. 1C). The stimulatory effect of lucigenin on NADPH consumption in microglial cells was nearly abolished by pre-treatment with DPI.

MAPK activation plays an essential role in the macrophage response to pro-inflammatory stimuli such as LPS and cytokines [35-37]. Therefore, we investigated whether ERK1/2 or p38 is activated in response to s-Mtb in BV-2 microglial or primary mixed glial cells. LPS induced p38 phosphorylation within 60 min of treatment. However, LPS did not stimulate ERK1/2 activation in BV-2 cells, indicating that ERK1/2 activation is not involved in LPS action in this cell type, which is consistent with previous finding [38]. S-Mtb stimulation activated both ERK1/2 and p38 in BV-2 cells. S-Mtb induced the phosphorylation of ERK1/2 and p38 within 5 min, and peak activity was observed after 15 min (Fig. 1D). Similarly, s-Mtb induced the phosphorylation of ERK1/2 and p38 in primary cultures of mixed glial cells. These results show that s-Mtb strongly induces NADPH oxidase-dependent ROS generation and activates MAPK signaling in microglia.

### S-Mtb stimulation induces pro-inflammatory cytokine production in murine microglia

**Figure 2:**
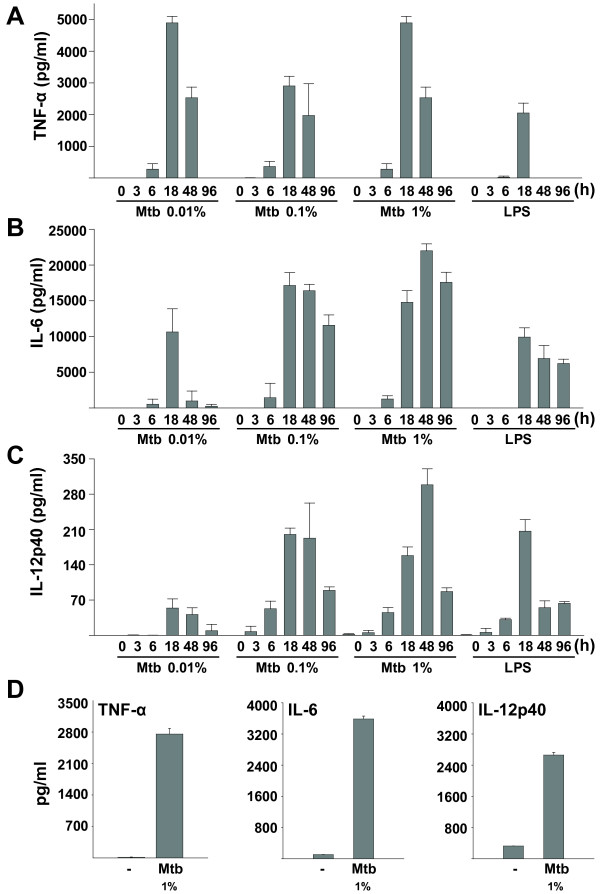
**S-Mtb increases pro-inflammatory cytokine production in microglia**. *A-C) *BV-2 cells were stimulated with 0.01, 0.1, or 1% s-Mtb or with 1 μg/ml LPS for the indicated times (0- 96 h). The supernatant was analyzed for cytokine production using ELISA. A significant increase in TNF-α (*A*), IL-6 (*B*), and IL-12p40 (*C*) production was observed. *D) *TNF-α, IL-6, and IL-12p40 protein levels in primary cultures of mixed glial cells were analyzed after 18 h of s-Mtb stimulation. Values are the mean ± SD of triplicate samples.

We examined the microglial production of pro-inflammatory cytokines in response to s-Mtb. Cell cultures were stimulated with different doses of s-Mtb (0.01, 0.1, or 1%), and the supernatant was collected at the indicated intervals for cytokine analysis. S-Mtb-stimulated BV-2 microglial cells produced robust amounts of TNF-α, IL-6, and IL-12p40 in a dose-dependent manner (Fig. 2A–C). Each cytokine had its peak response at 18 h or 48 h, which declined with prolonged treatment. LPS also induced cytokine production, although to a lesser extent than s-Mtb. Cytokine production in primary cultures of mixed glial cells was observed after 18 h of s-Mtb stimulation (Fig. 2D).

### The ERK1/2 and p38 pathways are critical for the s-Mtb-induced production of TNF-α, IL-6, and IL-12p40 in murine microglia

**Figure 3:**
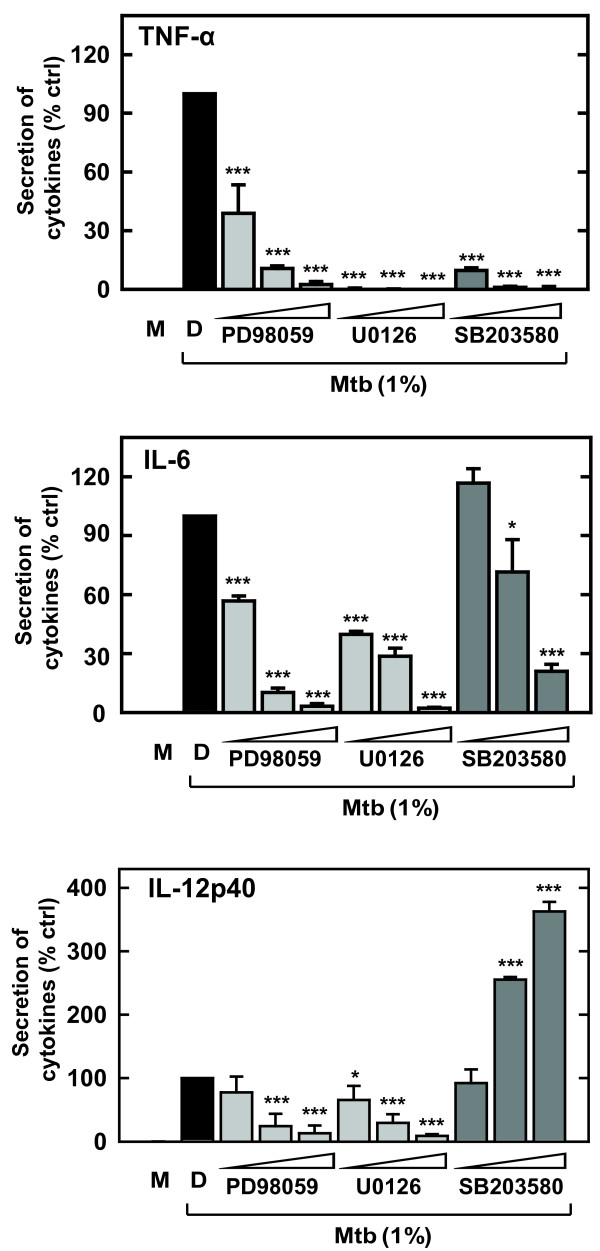
**Effect of MAPK inhibitors on pro-inflammatory cytokine production in s-Mtb-stimulated microglia**. BV-2 cells were pre-incubated with or without protein kinase inhibitors (PB98059, 5, 10, or 20 μM; U0126, 5, 10, or 20 μM; SB203680, 1, 5, or 10 μM) for 30 min and then incubated with 1% s-Mtb for 18 h. The supernatant was analyzed for TNF-α, IL-6, and IL-12p40 by ELISA. Data are expressed as the mean ± SD of values from three separate experiments. Significant differences compared to cultures incubated with 1% s-Mtb alone: *, *P < 0.05*; ***, *P < 0.001*. M, media only; D, 0.1% DMSO.

To better understand the functional roles of the ERK1/2 and p38 pathways in the s-Mtb-induced pro-inflammatory response, we assayed cytokine production in the absence or presence of specific inhibitors of ERK1/2 and p38. Pre-treatment with the MEK inhibitors PD98059 and U0126 or the p38 inhibitor SB203580 prevented s-Mtb-induced TNF-α and IL-6 production in BV-2 microglial cells in a dose-dependent manner (Fig. 3). Similarly, IL-12p40 production was inhibited in the presence of PD98059 and U0126. In contrast, IL-12p40 production was significantly up-regulated by SB203580 in a dose-dependent manner. These data indicate that the ERK1/2 and p38 pathways positively regulate TNF-α and IL-6 production, whereas the p38 pathway negatively regulates s-Mtb-induced IL-12p40 production in microglia.

### Intracellular ROS formation is essential for MAPK activation and pro-inflammatory cytokine production

**Figure 4:**
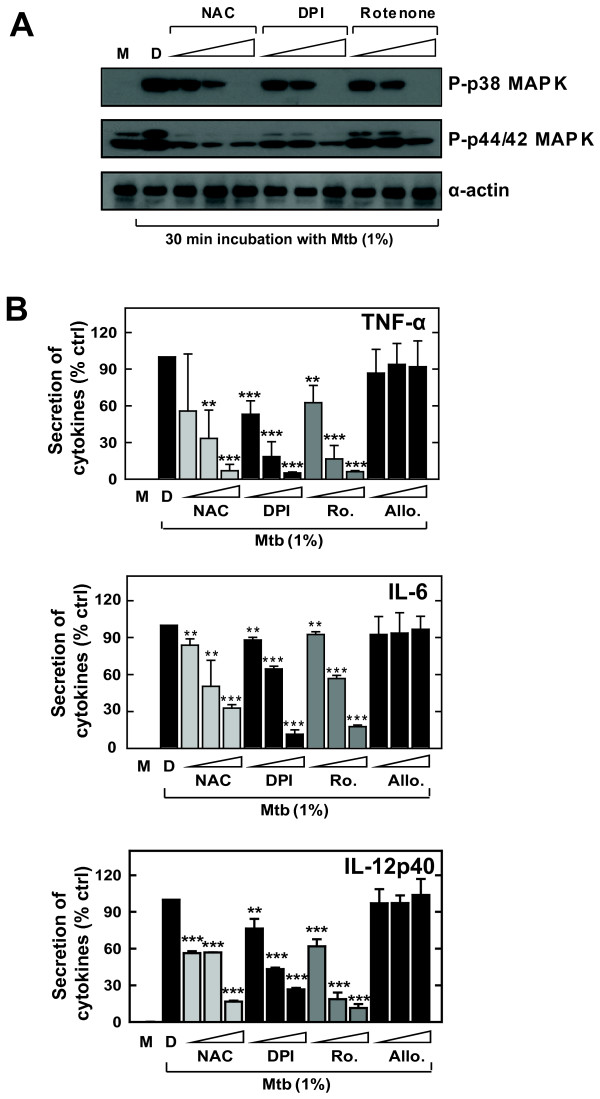
**ROS scavengers block MAPK activation and pro-inflammatory cytokine production in s-Mtb-stimulated microglia**. *A) *Effect of ROS scavengers on p38 and ERK1/2 activation. After pre-treatment for 30 min with NAC (10, 20, or 30 mM), DPI (10, 20, or 50 μM), or rotenone (1, 10, or 100 μM), BV-2 cells were stimulated with 1% s-Mtb for 30 min. The cells were then harvested and subjected to Western blot analysis to detect phosphorylated p38 and ERK1/2. The same blots were washed and blotted for α-actin as a loading control. *B) *Effect of ROS scavengers on TNF-α, IL-6, and IL-12p40 production. Cells were pre-treated with NAC, DPI, and rotenone as described in *A*. Culture supernatants were harvested after stimulation with 1% s-Mtb for 18 h, and TNF-α, IL-6, and IL-12p40 expression was measured by ELISA. The mean amount ± SD of TNF-α, IL-6, or IL-12p40 from cultures treated with 1% s-Mtb or 0.1% DMSO was set at 100, and the relative drop in cytokine production in the presence of each inhibitor is indicated. Significant differences compared to cultures incubated with 1% s-Mtb alone: **, *P *< 0.01; ***, *P *< 0.001. M, media only; D, 0.1% DMSO as a solvent control.

We examined whether intracellular ROS formation plays a role in MAPK activation and cytokine release in microglia using various inhibitors of ROS generation. As shown in Fig. 4A, S-Mtb-induced ERK1/2 and p38 activity in BV-2 microglial cells was substantially attenuated in the presence of such ROS scavengers as NAC (a general ROS scavenger), DPI (an NADPH oxidase inhibitor), and rotenone (a mitochondrial electron transfer chain subunit I inhibitor) in a concentration-dependent manner.

To evaluate whether ROS are involved in s-Mtb-mediated pro-inflammatory cytokine production, BV-2 microglial cells were pre-treated with various ROS scavengers. Pre-treatment with NAC, DPI, or rotenone significantly attenuated s-Mtb-induced TNF-α, IL-6, and IL-12p40 production in microglia (Fig. 4B). In contrast, pre-treatment with allopurinol, a xanthine oxidase inhibitor, did not affect MAPK activation or cytokine production in microglia (Fig. 4B, MAPK activation data not shown). These data suggest that s-Mtb-induced MAPK activation and pro-inflammatory cytokine release in microglial cells are probably mediated via ROS generated by NADPH oxidase and mitochondria.

### Activation of the cytosolic NADPH oxidase component p47phox and MAPK is mutually dependent on s-Mtb-induced inflammatory signaling in murine microglia

**Figure 5:**
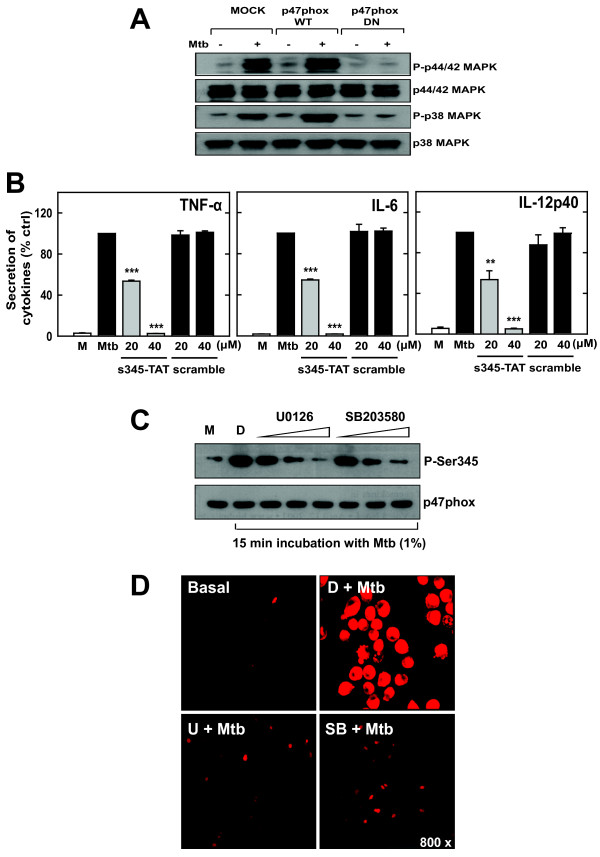
**The cytosolic NADPH oxidase subunit p47phox and MAPK activation is mutually dependent on the ROS generation and cytokine production by s-Mtb-stimulated microglia**. *A) *p47phox is required for s-Mtb-induced MAPK activation. BV-2 cells were transfected with wild-type p47phox (WT), dominant-negative p47phox (DN), or empty vector. The cells were then stimulated with 1% s-Mtb for 30 min, harvested, and subjected to Western blot analysis to detect total and phosphorylated ERK1/2 and p38. *B) *p47phox phosphorylation at Ser345 is required for s-Mtb-induced cytokine production. BV-2 cells were pre-treated with TAT-Ser345 peptide (20 or 40 μM) or TAT-scramble peptide (20 or 40 μM) and stimulated with 1% s-Mtb for 18 h. The supernatants were analyzed for TNF-α, IL-6, and IL-12p40 production by ELISA. Data are presented as the percentage of the control. Significant differences compared to cultures incubated with s-Mtb alone: **, *P *< 0.01; ***, *P *< 0.001. *C*) MAPK activation is essential for p47phox activation. After pretreatment for 30 min with inhibitors of either MEK1 (U0126; 5, 10, or 20 μM) or p38 (SB203580; 1, 5, or 10 μM), BV-2 cells were stimulated with 1% s-Mtb for 30 min. The cells were then harvested and subjected to Western blotting to detect phosphorylated pSer345 and p47phox. *D*) MAPK activity is required for s-Mtb-induced superoxide production. BV-2 cells were pretreated with U0126 (10 μM) or SB203580 (5 μM) for 30 min and then incubated with DHE (for superoxide detection) after stimulation with 1% s-Mtb for 30 min. The live cells were washed with serum-free medium and imaged using confocal microscopy. The images are representative of three independent experiments. M, medium only; D, 0.1% DMSO as a solvent control.

Phosphorylation of the cytosolic subunit p47phox is necessary for NADPH oxidase activation and regulation [39]. Although p47phox has been detected in cultured microglia [26], its role in MAPK activation and cytokine production in microglia has not been investigated. To examine whether ERK1/2 or p38 activation is dependent on p47phox activation, we examined the effect of wild-type (WT) or dominant-negative (DN) p47phox constructs on p38 and ERK1/2 phosphorylation. Our results showed that ERK1/2 and p38 phosphorylation increased substantially in BV-2 microglia transfected with WT p47phox, whereas phosphorylation was abolished in cells expressing DN p47phox (Fig. 5A). In addition, we pre-treated cells with an inhibitory cell-permeable peptide (TAT-Ser345 peptide) that corresponds to amino acids 339–350 (ARPGPQSPGSPL) of p47phox [40]. In cells treated with the TAT-Ser345 peptide, TNF-α, IL-6, and IL-12p40 production decreased significantly in a dose-dependent manner, whereas the TAT-scramble peptide had little or no inhibitory effect on cytokine production (Fig. 5B). These results suggest that p47phox activation is necessary for MAPK activation and the pro-inflammatory response in microglial cells.

It was reported that p47phox phosphorylation at Ser345 serves as a point of convergence for various MAPKs to induce the priming of ROS production [40]. To explore the possible role of MAPK upstream from the NADPH oxidase in microglia, we examined the effects of MAPKs inhibitors on the phosphorylation of p47phox and ROS production in BV2 microglial cells. Pretreatment with inhibitors of MEK1 (U0126) or p38 (SB203580) significantly downregulated the phosphorylation of p47phox in BV2 cells in a dose-dependent manner (Fig. 5C and 5D). In addition, superoxide production by BV-2 cells was substantially inhibited by pretreatment with inhibitors for MEK1 (U0126) and p38 (SB203580). Combined, these findings indicate that p47phox phosphorylation and MAPK (ERK1/2 and p38) activation are mutually dependent on s-Mtb-mediated inflammatory signaling pathways in microglial cells.

### Neither TLR2 nor dectin-1 is involved in s-Mtb-induced inflammatory mediator expression in murine microglia

**Figure 6:**
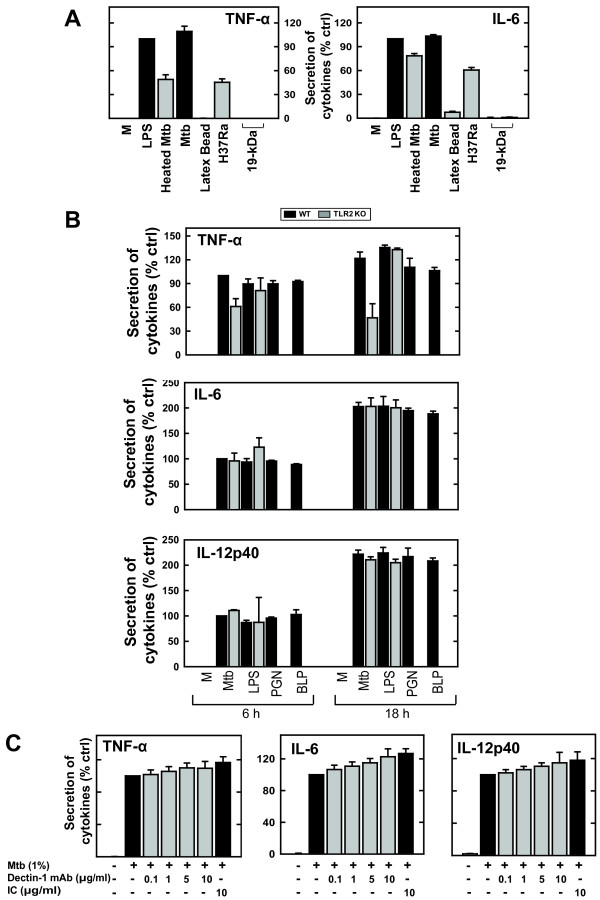
**S-Mtb-induced microglial activation is not associated with TLR2 or dectin-1**. *A) *Measurement of TNF-α and IL-6 from cultures stimulated with 1 μg/ml LPS, 1% s-Mtb, or 19-kDa (0.5 or 1 μg/ml). Latex beads, heated Mtb, and H37Ra were added with MOI at 1. The supernatants were harvested at 18 h and cytokine production was quantified by ELISA. Data are presented as the percentage of the control and compared to cultures incubated with LPS. *B) *Comparison of cytokine production in mixed glial cells from WT and TLR2 -/- mice. Primary mixed glial cells from WT and TLR2-/- mice were prepared and stimulated with 1% s-Mtb, 1 μg/ml LPS, 10 μg/ml PGN, or 10 nM BLP. The supernatants were harvested at 6 h and 18 h and analyzed for TNF-α, IL-6, and IL-12p40 production by ELISA. Data are presented as the percentage of the control and compared to cultures incubated with s-Mtb for 6 h. *C) *BV-2-cells were stimulated with 1% s-Mtb in the presence of PBS or a neutralizing monoclonal antibody against dectin-1. After 1 h of incubation at 37°C, the supernatants were analyzed for cytokine production by ELISA. Data are presented as the percentage of the control and compared to cultures incubated with s-Mtb alone. IC, isotype control.

Among the PRRs, TLR2 and dectin-1 are thought to be pivotal mediators of Mtb signaling. Thus, we investigated whether TLR2 or dectin-1 mediates s-Mtb-induced inflammatory cytokine production in microglia. S-Mtb, heat-denatured Mtb (H37Rv), and H37Ra induced TNF-α and IL-6 production, indicating that a heat-stable, non-protein bacterial component activates the pro-inflammatory response in microglial cells. Latex bead phagocytosis had no effect (Fig. 6A). Importantly, cytokine production in BV-2 microglial cells was not affected by treatment with 19-kDa antigen, which is a well-characterized mycobacterial TLR2 agonist. These data suggest that TLR2 may not be the only receptor that mediates the s-Mtb-induced pro-inflammatory response in microglia. Furthermore, we examined the expression of pro-inflammatory mediators in mixed glial cells from TLR2 -/- mice (Fig. 6B). Although the level of TNF-α was slightly lower in the TLR2 -/- cells than in WT cells, neither the TLR2 nor the dectin-1 blockade had an effect on the s-Mtb-induced pro-inflammatory response in microglia (Fig. 6B and 6C). Taken together, we conclude that neither TLR2 nor dectin-1 plays an indispensable role in s-Mtb-induced pro-inflammatory cytokine production in murine microglia; instead, s-Mtb appears to activate inflammatory responses via an as yet unknown PRR.

### Neither astrocytes nor indirect stimuli such as IL-1β adversely affected the s-Mtb-induced ROS release and cytokine production by primary mixed glial cells

**Figure 7:**
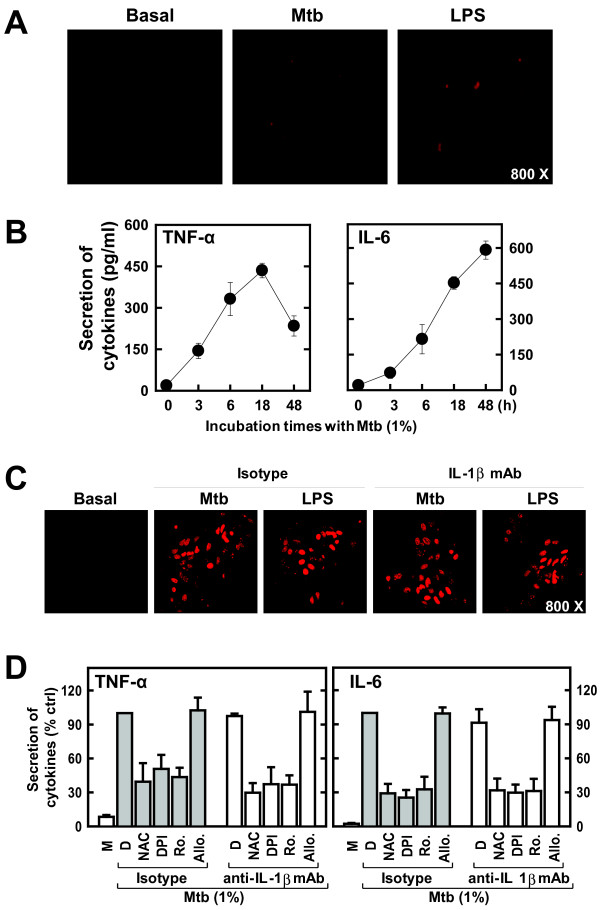
**Neither astrocytes nor indirect stimuli such as IL-1β adversely affected the s-Mtb-induced ROS release and cytokine production by primary mixed glial cells**. *A*) ROS generation in astrocyte-enriched cultures. Astrocyte-enriched cultures were incubated with DHE (for superoxide detection) in the presence or absence of 1% s-Mtb or 100 ng/ml LPS for 30 min. The live cells were washed with serum-free medium and imaged using confocal microscopy. Images are representative of three independent experiments. *B*) Cytokine production in astrocyte-enriched cultures. Astrocyte-enriched cultures were stimulated with 1% s-Mtb for the indicated times (0- 48 h). The supernatant was analyzed for cytokine production using ELISA for TNF-α and IL-6. Values are the mean ± SD of triplicate samples. *C*) The effects of IL-1β on superoxide production. Primary mixed glial cells were stimulated with 1% s-Mtb or 100 ng/ml LPS in the presence of a neutralizing monoclonal antibody to IL-1β (1 μg/ml) or isotype control (1 μg/ml). After a 1-h incubation at 37°C, the cells were incubated with DHE (for superoxide detection) for 30 min. The live cells were washed with serum-free medium and imaged using confocal microscopy. The images are representative of three independent experiments. *D*) The effects of IL-1β on cytokine production. Primary mixed glial cells were pre-incubated with a neutralizing monoclonal antibody to IL-1β (1 μg/ml) or isotype control (1 μg/ml) in the presence or absence of NAC (20 mM), DPI (20 μM), rotenone (10 μM), allopurinol (100 μM), or 0.1% DMSO control for 30 min. The cells were then stimulated with 1% s-Mtb for 18 h. The supernatant was analyzed for cytokine production using ELISA for TNF-α and IL-6. Values are the mean ± SD of triplicate samples.

To investigate the cellular sources of the s-Mtb-induced ROS and cytokines, astrocyte-enriched (> 95% astrocytes) cultures were collected and exposed to s-Mtb (1%). The intracellular ROS and cytokine production was then measured in these cell cultures. S-Mtb stimulation induced ROS generation, as well as TNF-α and IL-6 production, in astrocyte-enriched cultures (Fig. 7A and 7B). However, the amounts of superoxide in primary astrocyte-enriched cultures were negligible when compared with those in primary mixed glial cell cultures. In addition, the production of TNF-α/IL-6 from astrocyte-enriched cultures was not comparable to that of primary mixed cultures (Fig. 2 and 7B). Thus, the microglial cell population plays a dominant role in ROS generation and the inflammatory response to s-Mtb.

Because IL-1β affected ROS generation by astrocytes [41], we also investigated whether the s-Mtb-induced cytokine and ROS production by primary mixed glial cells resulted from indirect stimuli such as IL-1β. To investigate this hypothesis, we examined the cytokine and ROS production from primary mixed glial cells in the absence or presence of anti-IL-1β Ab (Fig. 7C and 7D). Both superoxide and H_2_O_2 _were robustly produced by primary mixed glial cells in response to s-Mtb, regardless of treatment with anti-IL-1β Ab (Fig. 7C and data not shown). In addition, s-Mtb-induced TNF-α and IL-6 production was not affected by pretreatment with anti-IL-1β Ab (Fig. 7D). Thus, neither astrocytes nor indirect stimuli such as IL-1β adversely affected the overall findings for primary mixed glial cells.

## Discussion

Given that human microglia are productively infected with Mtb and may be the principal cellular target in the CNS [4,5,42], understanding the molecular mechanisms of microglial activation and the anti-microbial response is required to develop targets for therapeutic intervention in CNS-TB. Rabbits are an excellent *in vivo *model for the study of CNS infection and pathogenesis because of their sensitive inflammatory response and their similarity to humans in terms of the clinical and histological symptoms of disease [43,44]. Mice are also used to study host immune responses to TB meningitis [45] because of the advantages in terms of genetic manipulation and the availability of commercial immunological reagents. We demonstrated that murine microglia produces pro-inflammatory cytokines in response to s-Mtb, and revealed the important roles of MAPK signaling and ROS production in this process. Although ROS signaling controls a broad range of physiological and pathological processes, including cellular proliferation, inflammation, and apoptosis [46,47], our study is the first to demonstrate its role in microglial activation in response to Mtb.

LPS activates both macrophages and microglial cells, which have specific roles in microbial defense within the peripheral and central nervous systems, respectively. Previously, Watters *et al*. [38] investigated the mechanism of LPS signaling in murine macrophages and microglial cells, and revealed different roles for MAPK signaling in these two cell types. We also demonstrated that LPS stimulates the production of TNF-α, IL-6, and IL-12p40 in murine BV-2 cells and in primary cultures of mixed glial cells, which is consistent with previous studies using primary cultures of human, murine, and rat microglial cells [5,48,49]. In contrast, very little research has been conducted regarding the mechanisms of recognition and intracellular signaling that induce the initial immune response to Mtb in microglia. In this study, we prepared non-infective Mtb lysates, as described previously by Netea *et al*. [31], and used them throughout the study. We found that s-Mtb strongly activated the inflammatory response and ROS generation in BV-2 microglial cell lines, as in those infected with live Mtb (data not shown). In addition, the astrocyte-enriched cultures did not play a major role in the s-Mtb-induced cytokine production and ROS generation by primary mixed glial cells. These findings are supported by previous findings that the tubercle bacillus preferentially infects human microglia, rather than astrocytes [5]. The same study also reported that microglial Mtb infection elicited the production of a variety of cytokines, including TNF-α, IL-1β, and IL-6 [5]. Because IL-1β affected the ROS generation from astrocytes [41] and because it might be released by activated microglia, we examined whether IL-1β affects the s-Mtb-induced ROS production by primary mixed glial cells. Pretreatment with anti-IL-1β Ab did not affect the s-Mtb-induced ROS generation or cytokine production, suggesting that the results for primary mixed glial cultures were specific to s-Mtb.

The mechanisms resulting in tissue destruction in TB meningitis are currently unclear. However, growing evidence suggests that inflammatory responses in the brain lead to tissue destruction in the distinct immunological setting of the CNS [50]. Roles for Mtb-induced proinflammatory cytokines and chemokines in CNS-TB have been suggested because dexamethasone treatment suppresses the production of pro-inflammatory cytokines and chemokines in Mtb-infected human microglia [5]. It may explain the beneficial effects of this adjunctive therapy with steroids on the outcome of TB meningitis [3]. Furthermore, recent studies by Harris *et al*. [51] showed that treating human astrocytes with conditioned medium from Mtb-infected monocytes significantly up-regulated matrix metalloproteinase-9, which suggests that Mtb may increase the activity of tissue-destructive matrix metalloproteinases. Therefore, pro-inflammatory mediators or tissue-destructive enzymes could contribute to the neurological damage observed in CNS-TB.

Our data clearly demonstrate that the activation of p47phox and MAPK is mutually dependent on inflammatory signaling in s-Mtb-stimulated microglia. The results indicate that ROS formation occurs immediately after s-Mtb stimulation and that ROS act as signaling molecules in MAPK activation and subsequent processes. Possible redox-dependent signaling leading to MAPK activation includes the H_2_O_2_-mediated inactivation of phosphatase and the deletion of the tensin homologue on chromosome 10 [52]. In addition, ROS mediate calcium release, as all three MAPKs are downstream of calcium-dependent processes [53]. These studies and our data suggest that the NADPH oxidase-derived ROS operate upstream of MAPKs. In addition, the data demonstrate that MAPK activation is required for the phosphorylation of p47phox and ROS production in microglial cells. The phosphorylation of p47phox at several serine residues within the polybasic region of the protein is an essential step in the activation of the NADPH oxidase complex [54]. Previous studies have shown that p47phox is a good *in vitro *substrate for ERK2 and p38 MAPK [55,56], and that the phosphorylation of p47phox on Ser345 is directly related to GM-CSF- and TNF-α-induced priming of ROS production [40]. Taken together, the crosstalk between p47phox and MAPK activation may play a pivotal role in the induction of ROS-dependent inflammatory responses by microglial cells.

Although they play different roles, both IL-12 and TNF-α are critical factors in the defense against mycobacteria. IL-12 is crucial for the differentiation of IFN-γ-producing Th1 cells [57]. Because mycobacteria are strong inducers of IL-12, mycobacterial infection can skew the response to a secondary antigen toward the Th1 phenotype [58]. We previously demonstrated that Mtb-induced IL-12 (p40 and p35) expression is negatively regulated by ERK1/2 signaling, whereas TNF-α is induced by ERK1/2 at both the transcriptional and translational levels in human monocyte-derived macrophages (MDMs) [13]. In the present study, we found that IL-12p40 was negatively regulated by p38, but not by ERK1/2. This is inconsistent with previous findings [59] showing that ERK1/2 suppresses the production of IL-12, whereas p38 promotes IL-12 production. This discrepancy may be the result of the differences between microglia and MDMs. Our results strongly suggest that macrophages and microglia have distinct regulatory machinery for the modulation of IL-12 proteins. Additional studies are required to clarify the precise regulatory mechanism of IL-12 production and its role in microglia.

It is important to identify the PRR that triggers microglial activation after Mtb stimulation. The TLR family recognizes a diverse spectrum of microbial ligands. TLR2 is classically recognized as a principal inducer of the pro-inflammatory signal, TNF-α, in response to Mtb [60]. In addition, it has been suggested that the soluble, heat-stable mycobacterial fraction signals mainly through TLR2, whereas the heat-labile components signal through TLR4 [61]. However, we showed that live, sonicated, or heated Mtb elicited robust amounts of cytokines in TLR2 knockout mixed glial cells, indicating that TLR2 is not essential for activation of the pro-inflammatory response. Our data also demonstrate that s-Mtb-induced pro-inflammatory cytokine production in microglia was not dependent on dectin-1. These results are partly consistent with previous studies [29] showing that TNF-α production in response to virulent *M. avium *724 and *M. tuberculosis *H37Rv was not dependent on dectin-1 in macrophages, although dectin-1 was required for TNF-α secretion in macrophages infected with *M. smegmatis *and other avirulent mycobacterial strains. Therefore, s-Mtb may be recognized through other PRRs or an as yet uncharacterized signaling pathway. To understand the neuropathogenesis of CNS-TB infection, additional studies are required to identify the PRRs that detect pathogen-derived molecules and lead to the development of both innate and adaptive immunity.

## Conclusion

In conclusion, our results provide important insight into microglial biology. First, s-Mtb is a potent inducer of ROS generation, pro-inflammatory cytokine production, and MAPK signaling. Second, intracellular ROS play an essential role in the regulation of s-Mtb-activated pro-inflammatory cytokine production in murine microglia, which is mediated via MAPK activation. Our data also emphasize the key roles of crosstalk between p47phox and MAPK activation in the pro-inflammatory response to s-Mtb in microglia.

## Abbreviations

BLP: Bacterial lipopeptide

CNS: Central nervous system

DHE: Dihydroethidium

DPI: Diphenyleneiodium

ELISA: Enzyme-linked immunosorbent assay

ERK: Extracellular signal-regulated kinase

H_2_DCFDA: 2',7'-Dichlorodihydrofluorescein diacetate

IL: Interleukin

LPS: Lipopolysaccharide

MAPKs: mitogen-activated protein kinases

Mtb: *Mycobacterium tuberculosis*NAC:*N*-acetyl-L-cysteine

NFκB: Nuclear factor kappa B

PCR: Polymerase chain reaction

PGN: Peptidoglycan

ROS: Reactive oxygen species

PRR: Pattern-recognition receptor

RT: Reverse transcription

TB: Tuberculosis

TLR: Toll-like receptor

TNF-α: Tumor necrosis factor-α

## Competing interests

The author(s) declare that they have no competing interests.

## Authors' contributions

CSY and HML performed the experiments on mouse primary mixed glial cells (cell cultures, ELISA, Western analysis) was involved in experimental design and interpretation of data, and reviewed the manuscript. JYL conducted experiments on intracellular ROS assay (intracellular ROS staining and NADPH oxidase), conceived the experiments, prepared the figures, and wrote the manuscript. DMS and JAK cultured the BV-2 cells and primary mixed glial cells, and performed the experiments on intracellular ROS assay. SJL conceived the experiments, obtained TLR2^-/- ^mice, and wrote the manuscript. DSL and YHL were involved in experimental design and interpretation of data. JE contributed vital new reagents and reviewed the manuscript. EKJ conceived the experiments, oversaw the project, prepared the figures and wrote the manuscript. All authors read and approved the final manuscript.
